# Scientific experiments on Tiangong-2, the predecessor of the China Space Station

**DOI:** 10.1093/nsr/nwac189

**Published:** 2022-09-07

**Authors:** He Zhu

**Affiliations:** Science and news editor at the editorial office of NSR in Beijing, China

## Abstract

‘The fact is that after ISS, China may be the only station in orbit,’ remarked Leroy Chiao, a former NASA astronaut and commander of the International Space Station (ISS). As of July 2022, Tianhe, the core module of the China Space Station (CSS), has been orbiting the Earth for more than a year, and the Wentian experiment module joined the Tianhe station on 25 July. Together, they make up about two thirds of the CSS. Later in October, the third piece of the puzzle, the Mengtian experiment module, will complete the T-shaped structure of the CSS. In 2024, the China Sky Survey Telescope, Xuntian, will enter orbit. The ISS, on the other hand, was just mentioned in Russia's response to the latest round of economic sanctions, confirming the concern expressed by Commander Chiao when he said ‘The ISS will be deorbited in 2030. The politics with the Russians is no longer tenable.’

As probably the only large platform for orbital experiments in the near future, Tianhe will host a large collection of exciting experiments in the coming years. These experiments cover a wide range of areas in fundamental physics, materials science, engineering and medicine. They were proposed and designed by Chinese and international scientists since Tianhe's predecessor, the Tiangong space lab, started the first round of orbital experiments in 2016. As the results of these experiments are gradually being published, we here explain and summarize the unique science behind them, for readers interested in research beyond conventional experiments conducted on Earth.

## COLD ATOMIC CLOCK

A fundamental requirement of many orbital experiments and deep space surveys is to accurately keep track of time. In modern physics, this important task is achieved with a cold atomic fountain clock. The atom commonly used on Earth is cesium (^133^Cs), which has a resonant frequency of 9.192 GHz. A microwave field at that frequency can excite low-energy (‘cold’) cesium atoms that later emit fluorescent light. Accurately tuning the microwave field to that frequency to maximize the fluorescent light will determine how accurately we can define 1 second. The general method of optimizing an atomic clock is to keep the microwave field as big as possible or move the cesium atoms in the microwave field as slowly as possible. Either approach will increase the time cesium atoms spend in the microwave field and increase the tuning accuracy. The operation of a cold atomic clock starts when one group of laser beams restricts the thermal motions of cesium atoms to form a small cloud and another group of laser beams tosses the cloud upward as in a water fountain. The path of the cesium cloud traveling up and down is where the microwave field is active. In this fountain-like design, making the field bigger would mean making the actual apparatus bigger, which is often not practical. The alternative is to reduce the velocity of the cesium cloud inside the microwave field. This velocity is the direct result of the gravitational pull from Earth. Therefore, conducting this experiment away from Earth where gravity is greatly reduced will directly result in more accurate tuning of the microwave field and a more accurate definition of time.

**Figure 1.**
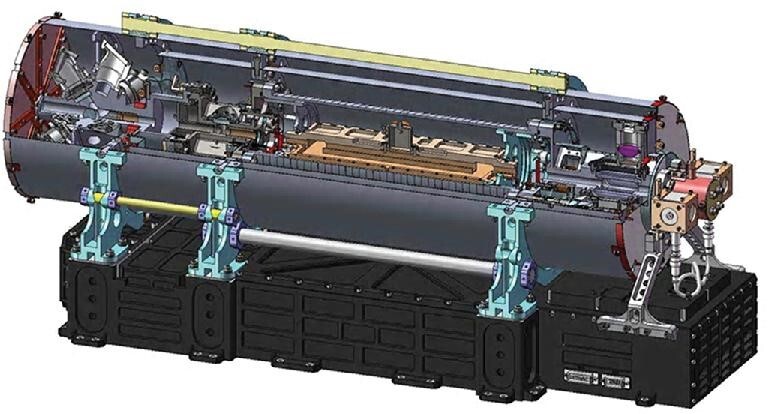
An illustration of the cold atomic clock used on the Tiangong-2 space laboratory. Provided by Dr. Liang Liu at Shanghai Institute of Optics and Fine Mechanics, CAS.

For orbital experiments conducted in Tiangong-2, the researchers led by Prof. Liang Liu at the Shanghai Institute of Optics and Fine Mechanics adopted an alternative design of the cold atomic fountain clock using ^87^Rb atoms at 6.834 GHz (Fig. 1). Compared to conventional cold atomic clocks on Earth, the orbital clock is more compact and consists of more elaborate shields against thermal and mechanical disturbances and electromagnetic waves. In addition, all operations of the clock are automated so adjustments and maintenance can be performed remotely. However, orbital operation presents several other issues not encountered on Earth. First, as the clock is orbiting the Earth, the magnetic field experienced by the clock is fluctuating and would degrade the accuracy of the clock if uncompensated. Therefore, a magnetic sensor monitors this fluctuation and adjusts the field generated by the clock itself in real time. With this correction mechanism, the variation of the magnetic field is limited to 4.5nT, which translates to a frequency instability of 1.7 × 10^−16^. Another issue unique to orbital operation arises from a region on Earth known as the South Atlantic Anomaly (SAA). This region encloses the majority of South America and part of the Atlantic Ocean to its east. For a reason that is still unknown, the magnetic field of the Earth in this region is lower than the rest of the world, so this region receives stronger cosmic rays and charged particles from space. When the orbital clock passes over this region, the photo luminescent detector registers random spikes in its signal. The operating software in the clock's control system identifies the locations of these spikes relative to the desired signal and removes them accordingly. As for the upgraded clock planned for the Tianhe space station, a more sophisticated and more powerful laser unit will be able to manipulate more ^87^Rb atoms to form a bigger cloud that in turn will produce more light signal in the luminescent detector. This will ultimately improve the accuracy of the clock even further. The details of the orbital atomic clock on the Tiangong-2 space lab were published in *Nature Communications* in 2018 [1] and *National Science Review* in 2020 [2].

## SOLIDIFICATION OF ALLOYS

**Figure 2.**
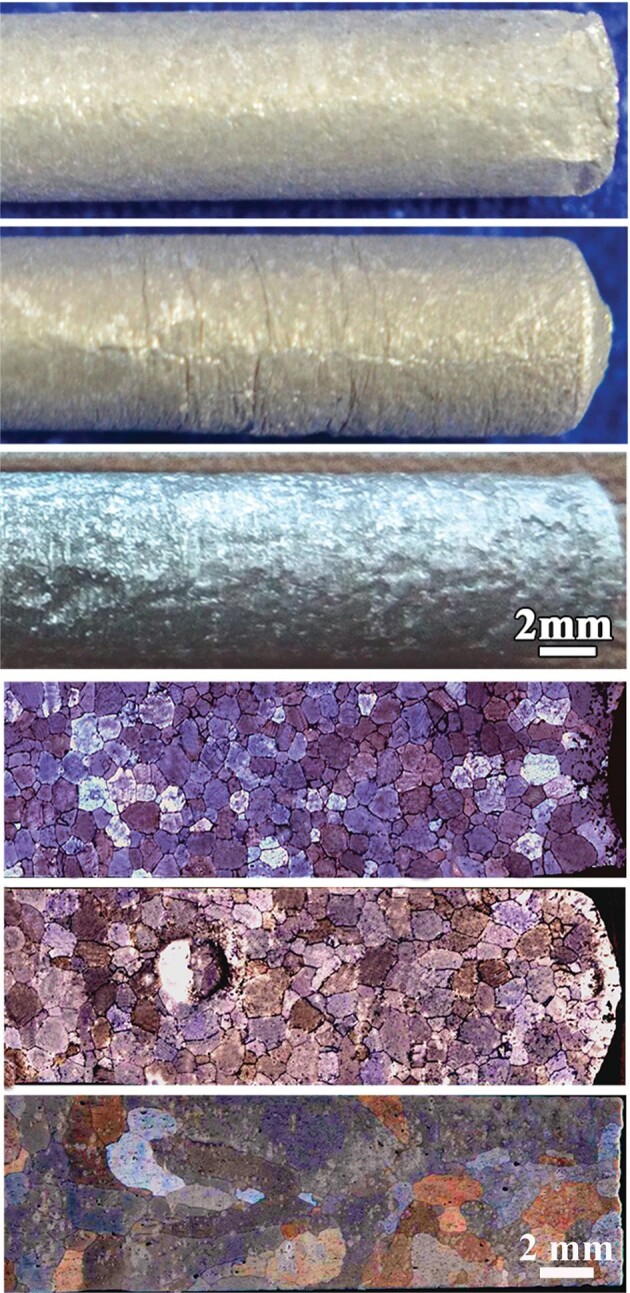
Samples of Al-Bi-Sn alloy. Top three: surface appearances of alloys formed in space, on Earth against gravity and on Earth along gravity (top to bottom). Bottom three: longitudinal sections in space, on Earth against gravity and on Earth along gravity (top to bottom). Images provided by Prof. Jiuzhou Zhao and originally published in *npj Microgravity* 2019; **5**: 26.

Phase transition has been a long-standing interest in materials science as it is essential in industrial applications such as semiconductors and alloys. In particular for alloys, the process of melting and solidification of two or more metal elements includes a special state when different types of liquid metals change between mixing and non-mixing. This special state, called the ‘miscible gap’, is where gravity can play a crucial role. When two or more liquid metals are in this complex state, the inherent density difference and temperature difference will cause the liquids to flow, known as convection. Solidification in the presence of convection determines the microstructure and mechanical properties of the formed solid. Scientists led by Prof. Jiuzhou Zhao at the Institute of Metal Research in Shenyang designed orbital experiments on alloys consisting of aluminum, bismuth and tin. The metals were heated to approximately 677°C in a small cylindrical container of 7 mm in diameter and 57 mm in length. The cooling process was achieved by pulling the container out of the heating components slowly in the course of approximately 2 hours. The procedure was repeated three times: once in space on the Tiangong-2 space lab, once on Earth with the container moving in the direction of gravity and once in the direction against gravity. The solidified alloy samples were then analyzed inside and out (Fig. 2). The sample formed in space showed a smooth surface while both samples formed on Earth showed rough patterns with many grooves. More importantly, when these samples were cut in half, the cross sections showed drastically different internal microstructures. The space sample showed finer and uniform grains while the Earth samples showed varying types of defects including bubbles, pinholes and large and irregular grains. The scientists also performed theoretical analysis in order to explain these crucial

differences. They concluded that microgravity in space resulted in much weaker convection flow when liquid metals turned into solids and that led to a denser and more uniform sample. As for the differences in terms of the alloy surfaces, that was believed to originate from the interaction between the liquid metals and the inner surface of the container. In microgravity, a liquid tends to cling to itself rather than to stick to the surface of the container. In many video recordings from space, astronauts demonstrated this with their drinking water. For liquid metals, the same phenomenon reduced the chance of surface defects caused by the container. The details of this series of experiments and its apparatus appeared in the journal *Microgravity* published by Nature Portfolio in 2019 [3].

## ROBOTIC ARMS

Efficient execution of orbital experiments is as important as their design especially when the environment is cumbersome and personnel are limited. To overcome this hurdle, the engineering team led by Prof. Hong Liu at the Harbin Institute of Technology developed two 7-joint, 6-DOF (degrees of freedom) robotic arms of lengths 5 m and 10 m. Each arm is equipped with a five-fingered dexterous hand, a hand-eye camera and a few environment cameras. Similar devices have been used on Earth in applications such as automotive assembly for decades. Translating this technology into space creates a few unique challenges. First, all the control programs and parameters designed to function on Earth naturally took into account a metallic arm's own weight, which is substantial. In a weightless environment, the control system needs to be updated entirely. Second, it is easy to imagine a frequent problem for astronauts, either inside a spacecraft or out: flying objects. It could be a screwdriver on the loose in the cabin or a piece of debris outside on its way to damage the spacecraft. So, a robotic arm needs to perfect the un-programmable task of catching something floating and spinning at the same time. For the first problem, the engineers developed a semi-autonomous teleoperation method. They first used a virtual reality system on Earth to generate training data for the robotic arm to perform a type of task, such as loosening a screw with an electric screwdriver. Then they relied on machine learning to let the robotic arm determine the specific commands for the joints and motors in the arm to achieve that task. Real-world tests of this system on Tiangong-2 demonstrated that the robotic arm can perform these tasks with smooth human-like motion, accurately and efficiently. As for the un-programmable task of object catching, the robotic arm relies on the images from the environment cameras as the primary input. As the control system issues commands to the components in the arm to move towards the object, it continuously predicts where the arm is in the process, based on the commands, the amount of gravity and the environmental disturbances such as vibration and air flow inside the spacecraft. This continuous prediction guides the control system in real time to optimize the commands being issued, until the path of the hand intersects with the path of the object and catches it. This control system was tested on Earth and in space onboard Tiangong-2 with successful results. The technical details of the task-learning and object-catching methods of the robotic arm were published in *IEEEAccess* in 2019 [4] and 2020 [5].

## Future experiments

The collection of experiments to be carried out onboard Tianhe will include nine international projects co-selected by the China Manned Space Agency (CMSA) and the United Nations Office for Outer Space Affairs (UNOOSA). For example, one experiment from the University of Oslo in Norway, smartly named ‘Tumors in Space’, will focus on how miniature organs of cancerous and healthy tissues grow differently under microgravity conditions. Such international collaborations may expand significantly as a second invitation will soon be issued by CMSA-UNOOSA to the global scientific community.
